# Supplementary material for: Transit amplifying cells coordinate mouse incisor mesenchymal stem cell activation
Source: Nat Commun. 2019 Aug 9;10:3596. doi: 10.1038/s41467-019-11611-0 (PMC6689115; doi:10.1038/s41467-019-11611-0)
Supplement: Supplementary file 1 — Supplementary Information [file 41467_2019_11611_MOESM1_ESM.pdf]

Supplementary Information

Transit Amplifying Cells Coordinate Mouse Incisor Mesenchymal Stem Cell Activation

Walker et al.,

Supplementary Figure 1

Supplementary Figure 2

Supplementary Figure 3

Supplementary Figure 4

Supplementary Figure 5

Supplementary Table 1

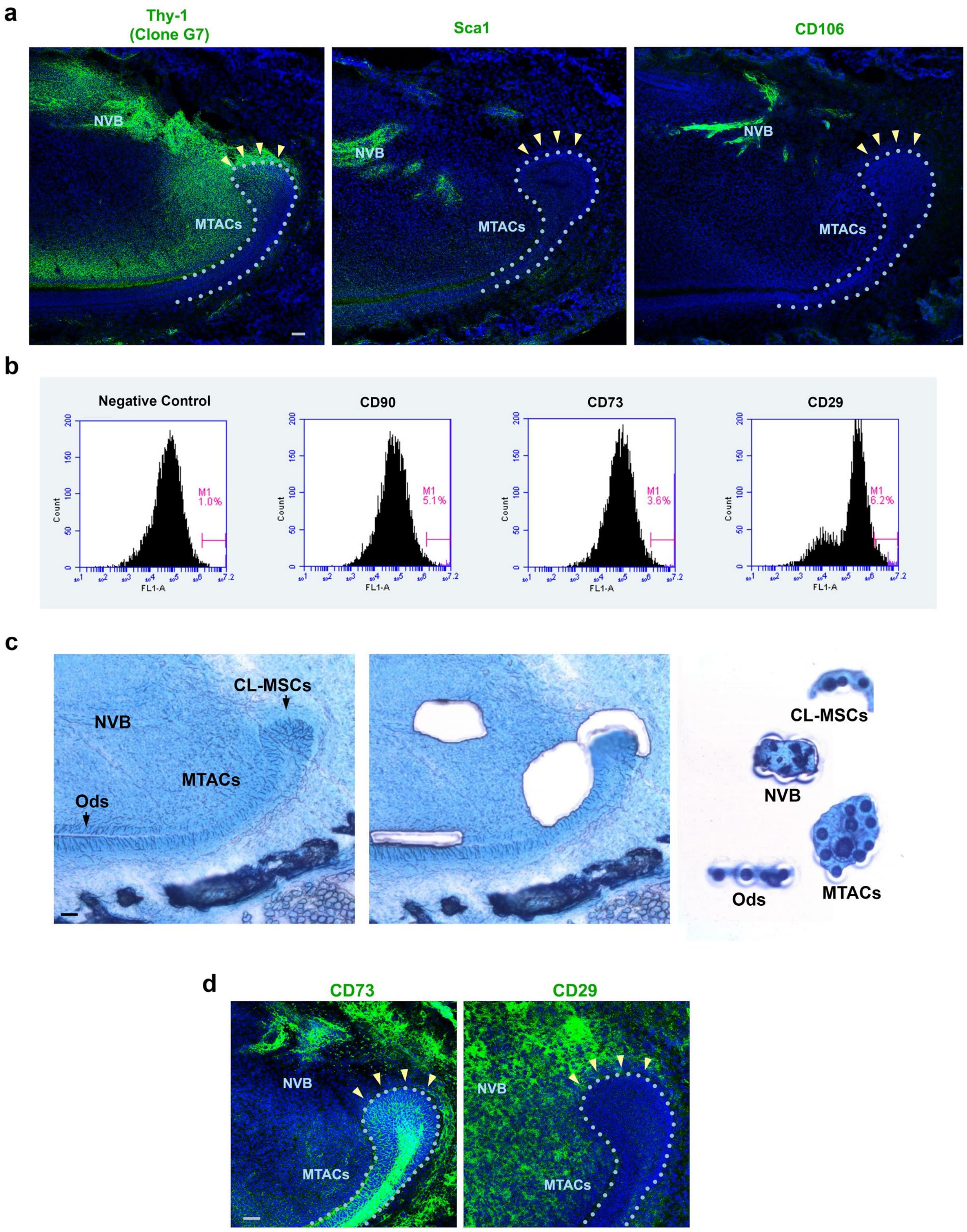

Supplementary Figure 1

Supplementary Fig. 1. Characterisations of mouse incisor CL-MSCs. **a** Immunofluorescence analysis of indicated markers using specific antibodies in P7 mouse incisors. Nuclei were counterstained with DAPI. Yellow arrowheads indicate the specific mesenchymal cells named as CL-MSCs. Light blue dotted line shows epithelial-mesenchymal junction. **b** Flow cytometry analysis of freshly enzymatically isolated CL-MSCs using CD90, CD73 and CD29 markers. The primary antibody was omitted in the negative control. Positive populations (M1) are indicated. **c** Illustration of laser capture microdissection technique used in the study. Tissue was stained with Methyl green prior dissection. CL-MSCs: cervical loop MSCs; MTACs: mesenchymal transit amplifying cells; NVB: neurovascular bundle region; Ods: Odontoblasts. **d** Immunofluorescent profiling of CD73 and CD29 in P7 mouse incisors. Nuclei were counterstained with DAPI. Note the presence of CD73 or CD29 positive cells in CL-MSC region. Yellow arrowheads indicate the specific mesenchymal cells named as CL-MSCs. Light blue dotted line shows epithelial-mesenchymal junction. Bar: **a** and **d**: 40μm **c**: 60μm.

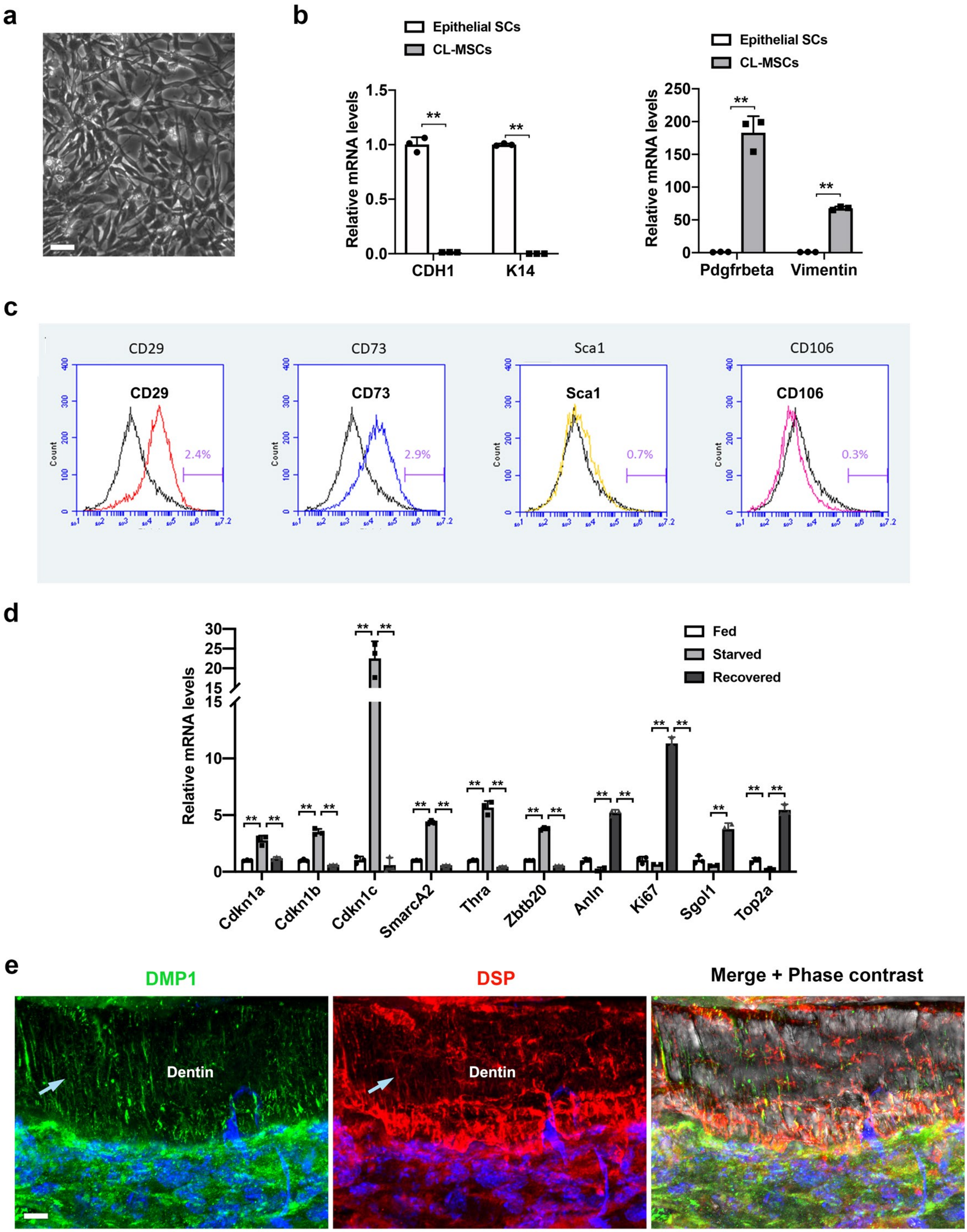

Supplementary Figure 2

Supplementary Fig. 2. CL-MSCs can be amplified *in vitro* and give rise to lineage differentiated cells. **a** A representative phase contrast image of CL-MSCs in culture after the second passage. **b** Mesenchymal and epithelial marker expression analysis of CL-MSCs at passage 2 (black) with epithelial cells (white) as the control. mRNA were extracted from n=3 biologically independent samples. Two-way ANOVA followed by Bonferoni correction was performed. Two asterisks: $p<0.01$. **c** Flow cytometry analysis of CD29, CD73, Sca1 and CD106 in CL-MSCs in culture for two passages. Primary antibody was omitted in negative control (black curve). Positive populations are indicated. **d** Real time RT-PCR analysis of the indicated markers in the cultured CL-MSCs under indicated conditions. mRNA were extracted from n=3 biologically independent samples. Two-way ANOVA followed by Bonferoni correction was performed. Two asterisks: $p<0.01$. **e** Immunofluorescence analysis of CL-MSCs (passage 4) growing on dentin slices for 5 days. Nuclei were counterstained with DAPI. Note the presence of DMP1- and DSP-positive fiber-like protrusions (arrows) penetrating into the dentin slice. Error bars represent standard deviation. Bar: **a** and **e**: 10μm.

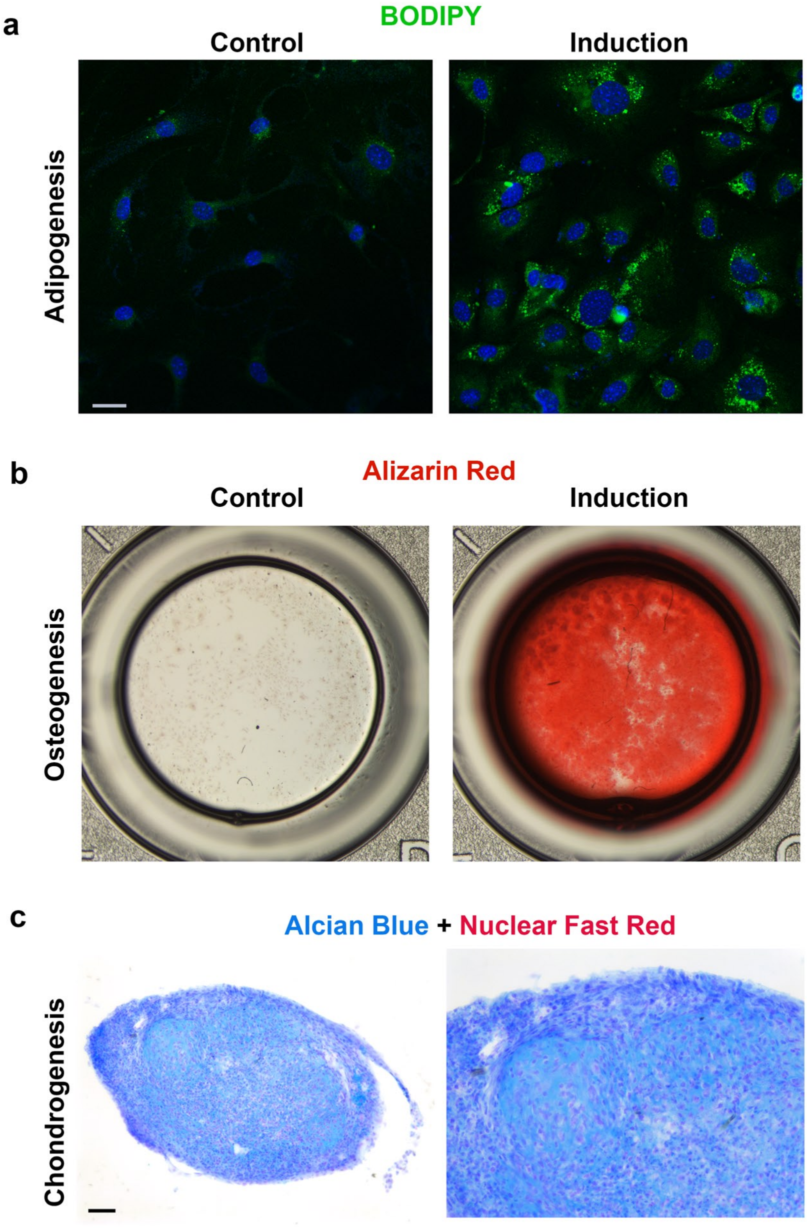

Supplementary Figure 3

Supplementary Fig. 3. CL-MSCs have multipotential differentiation abilities. **a-c** CL-MSCs at passage 1 were induced for adipocyte: **a**, osteoblasts: **b** and chondrocyte: **c** differentiation using the appropriate medium, and processed for BODIPY, Alizarin Red and Alcian Blue/Nuclear Fast Red labelling, respectively. As control, cells were maintained in normal medium. Micrographs showing typical data from triplicates obtained after 2-3 weeks' induction. Bars: **a**: 10μm; **c**: 50μm.

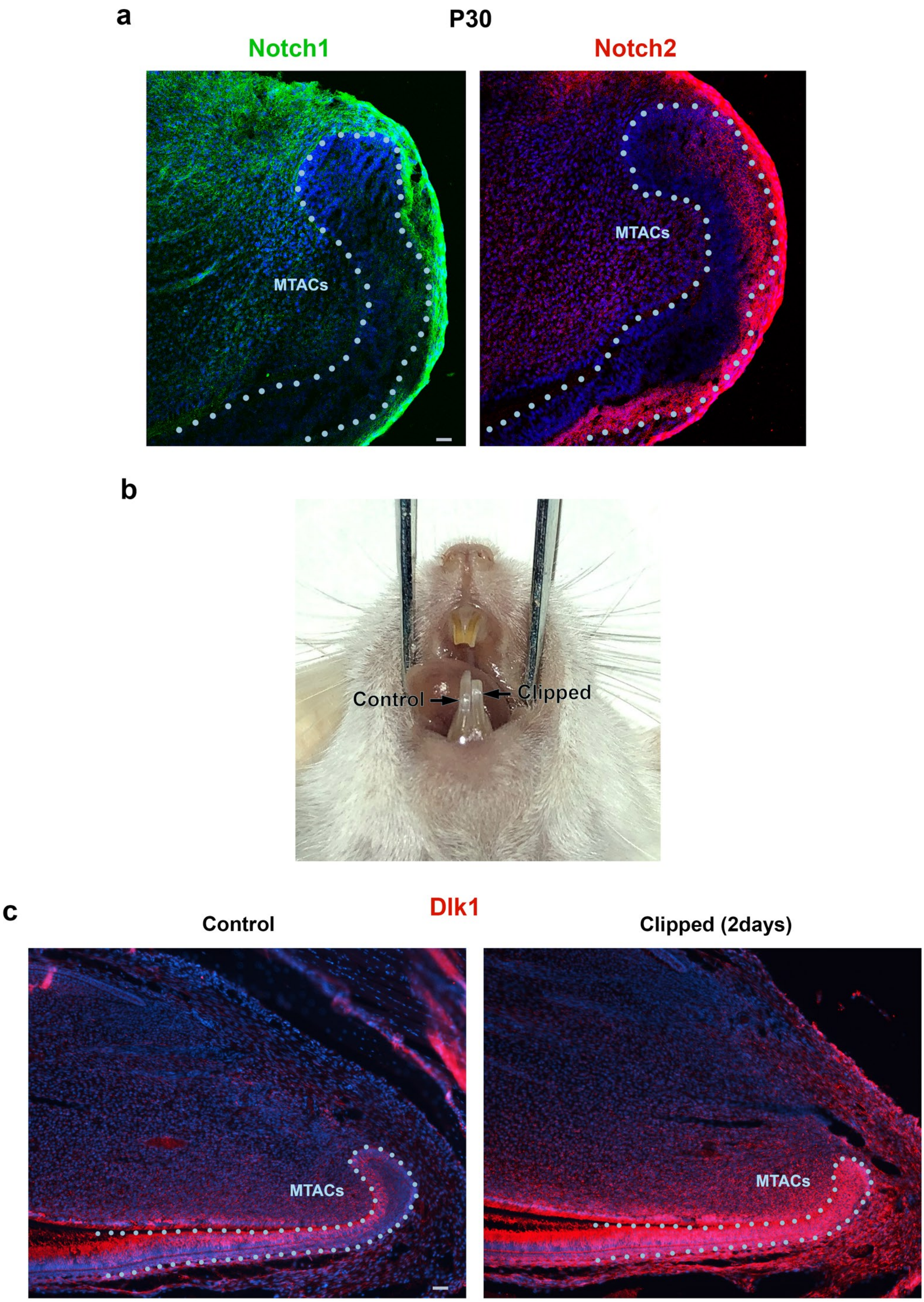

Supplementary Figure 4

Supplementary Fig. 4. Dlk1 expression was induced at CL after mouse lower molar clipping. **a** Immunofluorescence analysis of Notch1 and Notch2 expression at CL at CD1 P30 mouse lower incisors. Light blue dotted line shows epithelial-mesenchymal junction. **b** Representative picture showing the mouse incisor clipping experiments. Note the right clipped incisor grew faster than the left control. Black arrows indicate the marked original gingival lines for both incisors. **c** Representative Dlk1 immunostaining results on a pair of incisors for the clipping experiments at CL region. Nuclei were counterstained with DAPI. Light blue dotted line indicates epithelial-mesenchymal junction. Note the strong induction of Dlk1 expression in the clipped incisor's MTACs. Bars: **a**: 10μm; **c**: 25μm.

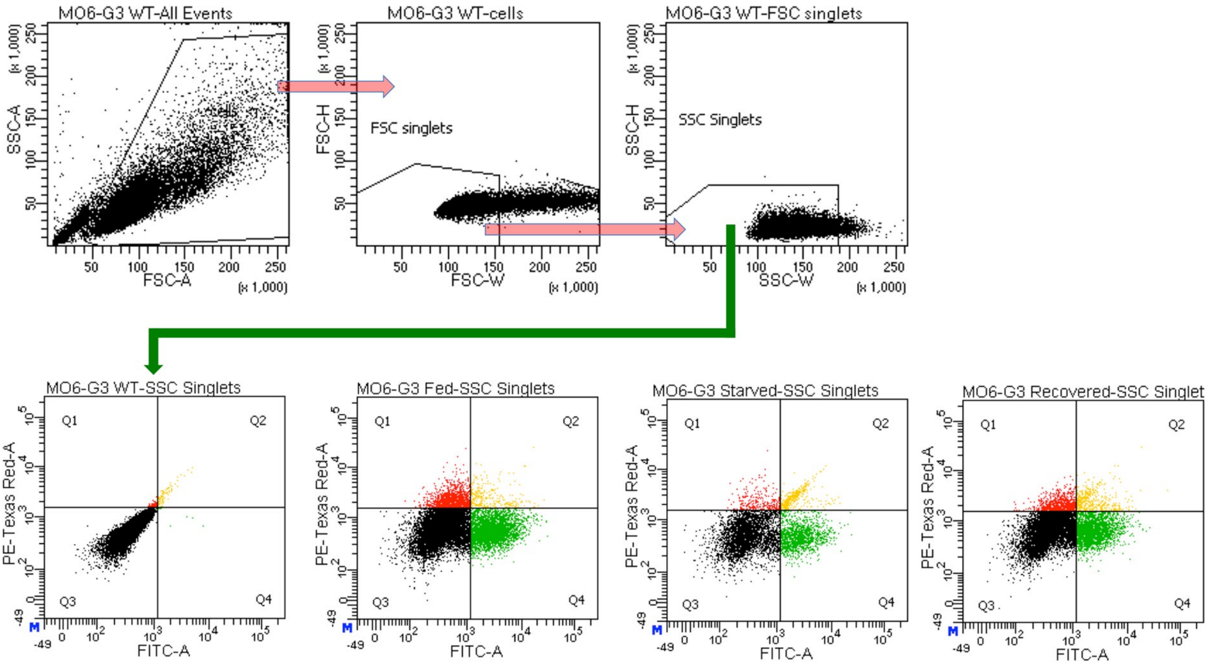

Supplementary Figure 5

Supplementary Fig. 5. Gating strategies used for in this study (see Figure 7f). Mouse incisor MSCs incorporated with Ki67p-T2A-FUCCI lentivirus were sorted based on green and red fluorescence.

Supplementary Table 1 **Antibodies and primers**

Primary antibodies				
**Target**	**Flow/Immunofluorescence/ChIP (Diluting factor)**	**Company**	**Cat. No.**	**Validation**
CD106	1:50 (Flow)	R&D Systems	MAB6432	PMID: 27552248
CD29	1:50 (Flow)	R&D Systems	MAB2405	PMID: 30016973
CD73	1:50 (Flow)	R&D Systems	MAB4488	Chinese hamster ovary cell line CHO-derived recombinant mouse 5'-Nucleotidase/CD73 Trp29-Lys549 Accession # Q61503
CD90 (Thy1)	1:50 (Flow)	R&D Systems	AF7335	PMID: 24562309
Dlk1	1:100 (IF)	Abcam	21682	PMID: 18556507; 19661059; 20331356; 22069189
Dll4	1:100 (IF)	R&D Systems	MAB1389	PMID: 25502884; 27898394
Dmp1	1:100 (IF)	R&D Systems	AF4386	PMID: 25551381; 24085820
Dsp	1:100 (IF)	Larry Fisher	LF-153	Validated by the provider
GFP	1:500 (IF)	Abcam	Ab290	One of the most widely used anti-GFP antibody
Gli1	1:200 (IF)	Novus	NBP1-78259	PMID: 24506883; 25799059
H3k27me3	1:200 (IF)	diagenode	pAb-056-050	PMID: 22581778; 23703216
H3k27me3	1μg (ChIP)	diagenode	pAb-056-050	PMID: 22581778; 23703216
H3k9me3	1:200 (IF)	diagenode	pAb-069-050	PMID: 28827334; 28031467
H3k9me3	1μg (ChIP)	diagenode	pAb-069-050	PMID: 28827334; 28031467
Hes1	1:100 (IF)	cell signalling	11988s	PMID: 25961456; 26512112
Ki67	1:200 (IF)	R&D Systems	AF7649	PMID: 24412617; 28300057
Notch 1	1:100 (IF)	eBioscience	14-5785	PMID: 29620159; 22682244
Notch 1 ICD	1:100 (IF)	cell signalling	4380	PMID: 30021650; 27849611
Notch 2 ICD	1:100 (IF)	cell signalling	5732	PMID: 26062937; 28084316
Notch 3	1:100 (IF)	R&D Systems	AF1308	PMID: 20960513; 29934496
PDGFRb	1:200 (IF)	Thermo Fisher Scientific	MA5-15143	PMID: 26304833; 26721734
Rbp Jk	100ng (ChIP)	cell signalling	5313	PMID: 27834400; 27312418
Sca1	1:50 (IF)	eBioscience	14-5981-82	PMID: 29934585; 29255233
SmarcA2	1:100 (IF)	Abcam	15597	PMID: 27044474; 22318225
CD90/Thy1-FITC (Clone 30-H2)	1:200 (IF)	eBioscience	11-0903-82	PMID: 29371677
CD90/Thy1 (Glone G7)	1:200 (IF)	eBioscience	14-0901-82	PMID: 29401595
Zbtb20	1:50 (IF)	sigma	HPA016815	PMID: 22318225; 29779894

Secondary antibodies			
**Target**	**Dilution (for immunolabelling)**	**Company**	**Cat. No.**
anti-goat Alexa Fluor 488	1/300	Life Technologies	A11055
anti-mouse Alexa Fluor 488	1/300	Life Technologies	A21202
anti-rabbit Alexa Fluor 568	1/500	Life Technologies	A10042
anti-rat Alexa Fluor 488	1/300	Life Technologies	A21208
anti-sheep Alexa Fluor 488	1/300	Life Technologies	A11015
Biotin IgG	1/200	VectorLab	BA-1100

Primers			
**Gene name**	**Forward primer ('5-'3)**	**Reverse primer ('5-'3)**	**Product Size**
Anln	ATTTTGTGCAAGACGCAACA	ATCCAGAGATCCCGCTCTTC	153
Ccna2	CATTGGCACAACAGACTGGA	TGTCTCTGGTGGGTTGAGAA	164
Ccnb1	TTCCTGTTATGCAGCACCTG	CGGCCTTAGACAAATTCTGA	159
Ccnd3	GCGATGTATCCTCCATCCAT	GCAGCTTCGATCTGTTCCTG	164
Cdh1	CCTGCCAATCCTGATGAAAT	GCCCCACTCGTTCAGATAAT	198
Cdkn1a	CTTGTCGCTGTCTTGCACTC	TCTCTTGCAGAAGACCAATCTG	142
Cdkn1b	TTGGGTCTCAGGCAAACTCT	TCTGTTGGCCCTTTTGTTTT	145
Cdkn1c	TCTAGGGGAATGGTTGTTGA	GATTTTTGTTGGGCCTCTTT	146
Ctdsp1	CGCCTCCTATGTCTTCCATC	GCCTAGGCTGTCTGAGCACT	149
Dlk1	GCGTGGACCTGGAGAAAG	GGAAGTCACCCCCGATGT	276
Dmp1	ACTGTCATTCTCCTTGTG	TTCATCATCCTCCTTATCG	300
Dspp	AGCATGTCCTTCTGGGAAGA	TCCTTTTGTCCAGCTCCACT	149
Ezh1	ACCCCAACTGTTATGCCAAA	CCTCTCGATGCCCACATACT	149
Foxo3	ACAAACGGCTCACTTTGTCC	CTGTGCAGGGACAGGTTGT	148
Hat1	TTCGACTGCTGGTGACTGAC	TGGTCTCAGGCATTTCTTCA	164
Hes1	CCAAGCTAGAGAAGGCAGACA	GTCACCTCGTTCATGCACTC	148
Hes5	AGGGTAGCAGCTTTCAGGAT	AGCCTCTGGGATCTCCTCTA	164
Hey1	GGTACCCAGTGCCTTTGAGA	ACCCCAAACTCCGATAGTCC	141
Hey2	TGCCAAGTTAGAAAAGGCTGA	AGCACTCTCGGAATCCAATG	137
Heyl	TTTGAGAAACAGGGCTCCTC	CCCAATACTCCGGAAGTCAA	141
K14	CCTGCTGGATGTGAAGACAA	ATCGTGCACATCCATGACCT	190
Ki67	AGCAAACCAGCTGCAGAAAT	TTCTTGGTGCATACAATGTCTT	194
Notch1	TGTTGTGCTCCTGAAGAACG	TCCATGTGATCCGTGATGTC	150
Notch2	GAGGCGACTCTTCTGCTGTT	CCATGTGGTCAGTGATGTCC	224
Pcna2	GAAGAGGAGGCGGTAACCAT	TGTCCCATGTCAGCAATTTT	167
PDGFRb	CAGAAATGCTGGGAAGAAAA	AACAGAGCTGGTGTCCAGAG	210
Pdk1	TACGGGACAGATGCGGTTAT	GGTCATGTCTTTCGGCTCTC	153
Ptov1	CGCCTGGTACAGTTCCACTT	TCCGAGGAGTACAGGAGCAT	145
Sgol1	CCAGCAGTGGCTCTGACTAA	TCATACCCTTTTCTGCTTGA	145
SmarcA2	AGGCGAAATCTGTGAAGGTG	TCAGTTCCACTTGCTTCTGACT	172
Tef	AGCTCTTCAACCCTCGGAAG	GAGCGTTTAGCTGCCACATT	147
Thra	GGCTGTGCTGCTAATGTCAA	TCACCTTCATCAGCAGCTTG	155
Top2a	GAACAGTCGCAAAAGGAAGC	GCTCGAGGAGCTATCGTGTC	147
Vimentin	CCAACCTTTTCTTCCCTGAA	GGTCATCGTGATGCTGAGAA	148
Zbtb20	CAAGGCGACAAAAAGCCTTA	GCGTCACCATGTGCTTGATA	169
Zfp30	GGCTGAGAAGAGAAGGTGGA	TTTCCCCAAAAGGGAGTCTT	154

Recombinant Proteins			
**Protein**	**Company**	**Cat. No.**	**Lot**
Human Dlk1	R&D	1144-PR/CF	HNE041302
Human Jagged 1	R&D	1277-JG-050	RZL1713041
Mouse Jagged 2	R&D	4748-JG-050	QXC1913101
