# Supplementary material for: Transit amplifying cells coordinate mouse incisor mesenchymal stem cell activation
Source: Nat Commun. 2019 Aug 9;10:3596. doi: 10.1038/s41467-019-11611-0 (PMC6689115; doi:10.1038/s41467-019-11611-0)
Supplement: Supplementary file 3 — Reporting Summary [file 41467_2019_11611_MOESM3_ESM.pdf]

nature research

Corresponding author(s): Dr Bing Hu

Last updated by author(s): Jun 24, 2019

Reporting Summary

Nature Research wishes to improve the reproducibility of the work that we publish. This form provides structure for consistency and transparency in reporting. For further information on Nature Research policies, see Authors & Referees and the Editorial Policy Checklist.

Statistics

For all statistical analyses, confirm that the following items are present in the figure legend, table legend, main text, or Methods section.

n/a	Confirmed	
☐	☒	The exact sample size (n) for each experimental group/condition, given as a discrete number and unit of measurement
☒	☐	A statement on whether measurements were taken from distinct samples or whether the same sample was measured repeatedly
☐	☒	The statistical test(s) used AND whether they are one- or two-sided<br>*Only common tests should be described solely by name; describe more complex techniques in the Methods section.*
☒	☐	A description of all covariates tested
☐	☒	A description of any assumptions or corrections, such as tests of normality and adjustment for multiple comparisons
☒	☐	A full description of the statistical parameters including central tendency (e.g. means) or other basic estimates (e.g. regression coefficient) AND variation (e.g. standard deviation) or associated estimates of uncertainty (e.g. confidence intervals)
☐	☒	For null hypothesis testing, the test statistic (e.g. F, t, r) with confidence intervals, effect sizes, degrees of freedom and P value noted<br>*Give P values as exact values whenever suitable.*
☒	☐	For Bayesian analysis, information on the choice of priors and Markov chain Monte Carlo settings
☒	☐	For hierarchical and complex designs, identification of the appropriate level for tests and full reporting of outcomes
☒	☐	Estimates of effect sizes (e.g. Cohen's d, Pearson's r), indicating how they were calculated

Our web collection on statistics for biologists contains articles on many of the points above.

Software and code

Policy information about availability of computer code

Data collection: *Provide a description of all commercial, open source and custom code used to collect the data in this study, specifying the version used OR state that no software was used.*

Data analysis: Graphpad Prism 6.0; Fiji 2.0; FlowJo; Imaris 9.0

For manuscripts utilizing custom algorithms or software that are central to the research but not yet described in published literature, software must be made available to editors/reviewers. We strongly encourage code deposition in a community repository (e.g. GitHub). See the Nature Research guidelines for submitting code & software for further information.

Data

Policy information about availability of data

All manuscripts must include a data availability statement. This statement should provide the following information, where applicable:

- Accession codes, unique identifiers, or web links for publicly available datasets
- A list of figures that have associated raw data
- A description of any restrictions on data availability

The datasets generated during and/or analysed during the current study are available from the corresponding author on reasonable request.

Field-specific reporting

Please select the one below that is the best fit for your research. If you are not sure, read the appropriate sections before making your selection.

☒ Life sciences ☐ Behavioural & social sciences ☐ Ecological, evolutionary & environmental sciences

Life sciences study design

All studies must disclose on these points even when the disclosure is negative.

Sample size	Animal sample size was calculated based on power of 80%
Data exclusions	No data exclusions in the study
Replication	Triplicates as standard for the experiments
Randomization	Full randomization was used
Blinding	Single blinding for animal experiments

Reporting for specific materials, systems and methods

We require information from authors about some types of materials, experimental systems and methods used in many studies. Here, indicate whether each material, system or method listed is relevant to your study. If you are not sure if a list item applies to your research, read the appropriate section before selecting a response.

Materials & experimental systems

n/a	Involved in the study	
☐	☒	Antibodies
☐	☒	Eukaryotic cell lines
☐	☐	Palaeontology
☐	☒	Animals and other organisms
☒	☐	Human research participants
☒	☐	Clinical data

Methods

n/a	Involved in the study	
☒	☐	ChIP-seq
☐	☒	Flow cytometry
☒	☐	MRI-based neuroimaging

Antibodies

Antibodies used	For details please see details in Supplementary Table 1
Validation	For details please see details in Supplementary Table 1

Eukaryotic cell lines

Policy information about cell lines

Cell line source(s)	Mouse incisor mesenchymal stem cells and human tooth pulp cells were prepared in the authors' labs. And MO6-G3 cells were achieved from University of Texas at San Antonio, originally developed by Prof. Mary MacDougall.
Authentication	MO6-G3 cells were achieved from the original developer's lab. Rest of the primary cells were not authenticated.
Mycoplasma contamination	All the cells were tested for mycroplasma contamination and no contamination were found.
Commonly misidentified lines (See ICLAC register)	no misindentified lines used

Palaeontology

Specimen provenance	*Provide provenance information for specimens and describe permits that were obtained for the work (including the name of the issuing authority, the date of issue, and any identifying information).*
Specimen deposition	*Indicate where the specimens have been deposited to permit free access by other researchers.*
Dating methods	*If new dates are provided, describe how they were obtained (e.g. collection, storage, sample pretreatment and measurement), where they were obtained (i.e. lab name), the calibration program and the protocol for quality assurance OR state that no new dates are provided.*

☐ Tick this box to confirm that the raw and calibrated dates are available in the paper or in Supplementary Information.

Animals and other organisms

Policy information about studies involving animals; ARRIVE guidelines recommended for reporting animal research

Laboratory animals	CD1 mice, Collagen1a2 Cre mice, RBP-Jkappa flox/flox mice, Transgenic Notch Reporter mice, Dlk1-/- mice, Collagen 1 α1 Dlk1 Tg mice, PDGFrbeta Cre ERT2 mice, ROSA mT/mG mice. Wistar rats.
Wild animals	*Provide details on animals observed in or captured in the field; report species, sex and age where possible. Describe how animals were caught and transported and what happened to captive animals after the study (if killed, explain why and describe method; if released, say where and when) OR state that the study did not involve wild animals.*
Field-collected samples	*For laboratory work with field-collected samples, describe all relevant parameters such as housing, maintenance, temperature, photoperiod and end-of-experiment protocol OR state that the study did not involve samples collected from the field.*
Ethics oversight	Ethic Committees at the authors' institutes, for details please see Methods section

Note that full information on the approval of the study protocol must also be provided in the manuscript.

Flow Cytometry

Plots

Confirm that:

☒ The axis labels state the marker and fluorochrome used (e.g. CD4-FITC).

☒ The axis scales are clearly visible. Include numbers along axes only for bottom left plot of group (a 'group' is an analysis of identical markers).

☒ All plots are contour plots with outliers or pseudocolor plots.

☒ A numerical value for number of cells or percentage (with statistics) is provided.

Methodology

Sample preparation	Cells were collected after digesting with TrypLE for 10-15 minutes then centrifugation at 1500 rpm for 5 min, to form single cell suspensions. After being resuspended, the single cells were analyzed using flow cytometer.
Instrument	BD FACSCanto II SOR (Beckman Coulter)
Software	Flowjo
Cell population abundance	>90% of the particles were single cells after the gating, which was verified by fluorescent microscopy in parallel.
Gating strategy	Cells were first gated on the basis of forward and side scatter properties, after which singlets were isolated on the basis of relationship between side scatter area peak area and width. A secondary-only negative control was used to determine the background fluorescence, and positive cells were quantified by setting a boundary so that less than 1% of the scondary-only control cells would be considered positive.

☒ Tick this box to confirm that a figure exemplifying the gating strategy is provided in the Supplementary Information.
